# Cooking Fuels in Lagos, Nigeria: Factors Associated with Household Choice of Kerosene or Liquefied Petroleum Gas (LPG)

**DOI:** 10.3390/ijerph15040641

**Published:** 2018-03-31

**Authors:** Obianuju B. Ozoh, Tochi J. Okwor, Olorunfemi Adetona, Ayesha O. Akinkugbe, Casmir E. Amadi, Christopher Esezobor, Olufunke O. Adeyeye, Oluwafemi Ojo, Vivian N. Nwude, Kevin Mortimer

**Affiliations:** 1 College of Medicine, University of Lagos, Idi-Araba, Lagos 100254, Nigeria; ahseya68@yahoo.com (A.O.A.); acetalx@yahoo.com (C.E.A.); esezobor@gmail.com (C.E.); 2 Lagos University Teaching Hospital, Idi-Araba, Lagos 100254, Nigeria; ojofemi911@yahoo.com (O.O.); ngozi_nwude@yahoo.com (V.N.N.); 3 University of Nigeria Teaching Hospital Ituku Ozalla, Enugu 400114, Nigeria; okwortochi@yahoo.com; 4 College of Public Health, Ohio State University, Columbus, OH 43210, USA; adetona.1@osu.edu; 5 College of Medicine, Lagos State University, Ikeja, Lagos 100271, Nigeria; olufunkeadeyeye@yahoo.com; 6 Liverpool School of Tropical Medicine, Liverpool L3 5QA, UK; Kevin.Mortimer@lstmed.ac.uk

**Keywords:** cooking fuels, kerosene, liquefied petroleum gas, attitudes and barriers

## Abstract

Cooking with dirty-burning fuels is associated with health risk from household air pollution. We assessed the prevalence of and factors associated with the use of cooking fuels, and attitudes and barriers towards use of liquefied petroleum gas (LPG). This was a cross-sectional, population-based survey conducted in 519 households in Lagos, Nigeria. We used a structured questionnaire to obtain information regarding choice of household cooking fuel and the attitudes towards the use of LPG. Kerosene was the most frequently used cooking fuel (*n* = 475, 91.5%; primary use *n* = 364, 70.1%) followed by charcoal (*n* = 159, 30.6%; primary use *n* = 88, 17%) and LPG (*n* = 86, 16.6%; primary use *n* = 63, 12.1%). Higher level of education, higher income and younger age were associated with LPG vs. kerosene use. Fuel expenditure on LPG was significantly lower than for kerosene (N (Naira) 2169.0 ± 1507.0 vs. N2581.6 ± 1407.5). Over 90% of non-LPG users were willing to switch to LPG but cited safety issues and high cost as potential barriers to switching. Our findings suggest that misinformation and beliefs regarding benefits, safety and cost of LPG are important barriers to LPG use. An educational intervention program could be a cost-effective approach to improve LPG adoption and should be formally addressed through a well-designed community-based intervention study.

## 1. Introduction

Household air pollution (HAP) is the single most important adverse environmental health risk factor globally and accounts for about 4.3 million premature deaths annually [1,2]. Most of these deaths occur in low and middle-income countries (LMIC) and are attributable primarily to pneumonia in children and non-communicable cardiorespiratory diseases in adults [1]. The use of dirty-burning cooking fuels that emit high levels of pollutants such as fine particulate matter (PM_2.5_) and carbon monoxide (CO) is a major source of HAP and are associated with exposure-related adverse health effects [3]. Cooking with kerosene, particularly in wick stoves produces levels of PM_2.5_, CO and other health damaging pollutants such as formaldehyde, polycyclic aromatic hydrocarbons, sulfur dioxide (SO_2_) and nitrogen oxides well above the World Health Organization (WHO) Air Quality Guideline (AQG) levels [4]. Kerosene is now considered a polluting fuel and WHO AQG recommends against its use [3]. Concentrations of PM_2.5_ from kerosene stoves in kitchens have been found to be 2–4 times higher than those for liquefied petroleum gas (LPG) stoves and this has been associated with many adverse effects such as increased risk of tuberculosis, low birth weight, chronic obstructive pulmonary disease, lower respiratory tract infections, lung cancer and lung function decline [5,6,7,8,9].

In Nigeria, there has been a steady transition from biomass to kerosene for cooking, and kerosene has been reported as the commonest cooking fuel in urban areas [10,11,12]. The Nigerian Demographic Health Survey (DHS) in 2013 reported that 26% of the population used kerosene for cooking comprising 48% and 9% of urban and rural households respectively [13]. This transition was initially driven by government subsidies on kerosene and energy sector influences which increased the affordability and availability of kerosene for a while [14]. However, kerosene subsidy was removed in 2016 which led to a near doubling of the pump price of kerosene and led to scarcity of the product. Many independent marketers could no longer afford to import the product and most kerosene users bought the product at about four times the government regulated price [15]. Motivations at individual household levels for the high rate of use of kerosene for cooking in Nigeria have not been extensively evaluated. This is even more pertinent at this time that kerosene subsidy has been removed, availability has reduced and compelling evidence on the potential harmful effects of kerosene have emerged [15,16]. Little health related research has been conducted on the use of kerosene fuel for cooking in Nigeria although there is a recent report of an association between cooking with kerosene and reduced lung function among women [9].

Based on the current evidence regarding the harmful health effects associated with kerosene use and in line with the 7th United Nations’ sustainable development goal (SDG) of affordable and clean energy for all, WHO recommends transition by kerosene users to cleaner cooking fuels such as liquefied petroleum gas (LPG) [17]. However, rates of transition away from kerosene to LPG remain low in Nigeria [15]. Understanding the factors associated with kerosene use and the attitude and barriers towards the use of LPG is central to the development of effective strategies that promote transition from kerosene to LPG. LPG is readily available and relatively affordable in urban areas in Nigeria, it is cleaner and more efficient than kerosene [18,19]. It is also a sustainable fuel for household cooking with expected health, social and economic benefits [18,19,20,21]. We assessed the prevalence of and factors associated with the use of kerosene, LPG, and other fuels for cooking in a densely populated community in Lagos, Nigeria. We also evaluated the attitude and barriers towards the use of LPG to provide useful insight for the design and implementation of interventions to scale up LPG adoption among kerosene users.

## 2. Materials and Methods

This was a cross-sectional, population-based study conducted in Mushin Local Government Area (LGA) of Lagos state, Nigeria. Lagos is the commercial capital of Nigeria and metropolitan Lagos is a densely populated city with a population density of a little over 18,000 per km^2^ [22]. The population in Metropolitan Lagos is about 14 Million (approximately 10% of the National population) and 85% of the population in Lagos state [22]. About 66% of the population in Lagos live in low income densely populated areas [23]. Mushin LGA, which is in the metropolis, is one of 20 LGAs in Lagos state. It is predominantly a low to middle income, urban area, with a population of approximately one and half million people according to the National Population Commission [23]. Mushin LGA is further sub divided into 21 communities administered by organized community development associations. For this study, we selected Idi-Araba community, which represents a densely populated area in the city of Lagos with a population of about 50,000 [24].

### 2.1. Ethical and Other Permissions

Following approval of the study protocol by the Lagos University Teaching Hospital Health Research Ethics Committee (ADM/DCST/HREC/APP/799), additional permissions were obtained from the community leaders. Notification of intended study date was disseminated to the community through the LGA representative about one week prior to the study commencement date to sensitize the inhabitants and improve response rate. All members of the community had equal opportunity to participate in the study. Written informed consent was obtained from individual participants.

### 2.2. Participant Selection

We used a multistage random sampling approach to select participants from each household. Idi-Araba community is clearly demarcated into four zones and in each zone, there are about ten streets. Each street has about 15 dwelling units and each typical dwelling unit is usually occupied by about 5–10 families, mostly in 1–2 room apartments with shared facilities such as bathrooms and kitchens. From each zone, we randomly selected five streets and from each street we selected ten dwelling units. All the households within each selected dwelling unit were eligible to participate. We included households in which an adult (18 years and above), who was either the primary cook or the primary decision maker regarding choice of household cooking fuel was present. From each selected household, we recruited and interviewed one member who met the inclusion criteria and was designated to provide the most reliable information about household cooking. This could be the male or female head of household, the wife in the household or other representative of the household.

### 2.3. Data Collection

The study was conducted over a 6 weeks period between June and July 2016. Trained interviewers administered a structured questionnaire to participants during a face to face interview. The interviewers were trained on the study objectives, how to obtain informed consent and how to administer the questionnaire to get valid responses. The questionnaire was pretested before use and adjusted as required prior to study onset. The questionnaire was administered in English or any of the local languages.

The structured questionnaire included open and close ended questions. We obtained socio-demographic information, household status of participants, role in decision making regarding choice of household cooking fuel, sources of current cooking fuel (indicating primary or main cooking fuel and secondary or substitute fuel), location of kitchen and previous primary fuel within the preceding five years if applicable. We assessed individual willingness and perception of community willingness to pay for LPG. Where the participant had changed primary fuel within five years or were unwilling to pay for LPG, we used open-ended questions to obtain the reasons for switching and unwillingness to pay respectively.

### 2.4. Data Management

Data was entered onto an excel sheet on a secure computer. All data was anonymized and coded as necessary in preparation for data analysis after checking for missing data. Missing data on household income (3%) and age (5%) occurred completely at random and was addressed statistically by average imputation prior to data analysis. We evaluated the responses obtained from open ended questions and categorized them into groups for analysis.

### 2.5. Sample Size Determination and Statistical Analysis

Based on anticipated frequency of kerosene use of 60% from on previous study [11], a minimum sample size of 361 participants/households was calculated to determine the frequency of kerosene use in this community with a 5% margin of error and 95% confidence.

Statistical analysis was performed using the Statistical Package for Social Sciences (SPSS) version 20 (IBM Corporation, Chicago, IL, USA). Continuous variables with normal distribution were expressed as means and standard deviation (SD) and compared using the student’s *t*-test or analysis of variance. Categorical variables were expressed as frequencies and compared using the chi square test. We explored the association between level of education, age, preparation of staple food and travel time to obtain fuel and primary fuel choice. We also compared the knowledge of the participants regarding the association between biomass and kerosene and specific disease conditions. We compared the knowledge of harmful health effects associated with kerosene use and biomass use and the influence on primary fuel choice. We categorized household income into multiples of the monthly minimum wage in Nigeria which is about N (Naira) 20,000 ($55) and obtained the monthly cost of cooking fuel across primary fuel choice. A *p*-value < 0.05 was considered significant for all comparisons.

## 3. Results

We recruited 519 participants (mean (SD) age 39.3 years (12.5); 81% female) most of whom were decision makers regarding choice of cooking fuel for their households (Table 1).

### 3.1. Choice of Household Fuel

Kerosene was the most frequently used cooking fuel among participants (*n* = 475, 91.5%) either as a primary or secondary cooking fuel, followed by charcoal and then LPG with limited use of either the dirtier (wood) or cleanest (electricity) fuels (Table 2). Fuel stacking whereby more than one fuel type was in use for cooking was common (*n* = 230, 44.3%). The median (interquartile range IQR) duration of use of primary cooking fuel was 10 (5–20) years.

The mother or female head of household prepared family meals in 86.9% of households and median time (IQR) spent cooking was 2 (1–2) h per day. Most primary kerosene users cooked outdoors or indoors separate from living and sleeping areas (Table 3). There was a significant difference in the location of kitchen among different fuel users with LPG users more likely to cook indoors in a kitchen separate from the living and sleeping area than users of other fuels (X^2^ = 26.7, *p* = 0.001).

### 3.2. Knowledge of Harmful Health Effects Associated with Kerosene Use Compared to Biomass Use

Knowledge of an association between kerosene and biomass (charcoal and wood) use respectively with specific disease condition (pneumonia, tuberculosis, breathing problems, lung cancer, asthma, bronchitis, running nose, watery eyes) was significantly higher for biomass than kerosene for all disease conditions (Table 4). Most participants (465, 89.6%) were aware of at least one harmful health effect associated with biomass use, but only (138, 26.6%) were aware of at least one harmful health effect associated with kerosene use (X^2^ = 14.0, *p* = 0.001).

### 3.3. Factors Associated with Choice of Household Cooking Fuel

There was a significant difference in the level of education among primary fuel users X^2^ = 52.2, *p* = 0.004 (Table 5). LPG and kerosene users were significantly more educated than charcoal users. Specifically, LPG users with post-secondary education were significantly higher than kerosene users with post-secondary education X^2^ = 17.0, *p* = 0.02.

None of the participants older than 60 years of age used LPG, electricity, or wood for cooking (Table 5). There was no significant difference in the age group among all primary fuel users (X^2^ = 24.7, *p* = 0.42) but when LPG users and kerosene users were compared alone, kerosene users tended to be older than LPG users (X^2^ = 13.6, *p* = 0.03).

More LPG users than kerosene and charcoal users earned more than N100,000 ($300) monthly, which is about five times the minimum wage in Nigeria (Table 5). The proportion of LPG users that earned less than N20,000 ($60) was similar to the proportion that earned over N100,000 ($300). The difference in household income among all primary fuel users was not significant (X^2^ = 40.8, *p* = 0.06), however, when only LPG users and kerosene users were compared, LPG users earned significantly more than kerosene users (X^2^ = 17.3, *p* = 0.02).

Rice was the staple food in 69.6% of households. The staple food in all households did not require any special cooking methods such as roasting or grilling. Method of preparation of staple food influenced fuel choice in 10.2% of all participants. There was a significant difference in the influence of the method of food preparation on fuel choice among different fuel users with more charcoal and LPG users reporting influence on fuel choice than kerosene users (X^2^ = 16.07, *p* = 0.04) (Table 5). Cooking time for staple food influenced choice in 10.2% of participants. Although more charcoal and LPG users reported influence of cooking time for staple food on fuel choice than other fuel users, the difference was not significant (X^2^ = 6.19, *p* = 0.19) (Table 5). Food taste, cultural method of cooking and specific method of cooking did not influence fuel choice among participants.

Most households (73%) irrespective of fuel type travelled <15 min to purchase fuel, 22% travelled 15 to 30 min and 5% travelled >30 min (Table 5). There was no significant difference in the time travelled to obtain cooking fuel among the fuel types (X^2^ = 12.73, *p* = 0.12).

There was a significant difference in monthly expenditure among fuel users (F = 1.58, *p* = 0.004) (Table 5). The monthly expenditure on fuel was highest for charcoal users and lowest for electricity users. Furthermore, the monthly expenditure for kerosene users was significantly higher than monthly expenditure for LPG (*t* = 2.13, *p* = 0.03).

Most participants across all fuel types knew at least one health effect associated with biomass use (Table 5). There was no significant difference in the knowledge of harmful health effect associated with biomass use among fuel users (X^2^ = 11.12, *p* = 0.20). More LPG users than kerosene users knew at least one harmful heath effect associated with kerosene use (Table 5). Charcoal users had the least knowledge of the harmful health effects associated with kerosene. The difference in the knowledge of harmful health effects associated with kerosene use among fuel users was not significant (X^2^ = 9.72, *p* = 0.05).

There was no significant difference in number in household, status in household, marital status, decision maker regarding household cooking fuel and main income earner or spender respectively among primary fuel users.

### 3.4. Factors Associated with Switching of Primary Household Cooking Fuel

Among primary kerosene users (*n* = 364), 11.2%, 2.7%, 1.9% and 1.6% had used charcoal, LPG, wood, and electricity, respectively, as primary fuel within the preceding 5 years. Among participants who switched to kerosene from charcoal or wood, health benefit associated with kerosene use was the reason for switching in 45.8%, while convenience, increase in income, high cost of charcoal or wood, marriage, and lack of space to use charcoal were given as reasons by 20.8%, 12.5%, 8.3%, 6.3% and 2%, respectively. No reason was provided for switching by 4.2%. Among those who switched from LPG to kerosene, cost of LPG was the reason for switching in 60% while fear of fire and harm to young children, restrictions by landlords, unavailability of LPG and convenience of obtaining and using kerosene were all given as reasons by 10% respectively. Inadequate power supply was the reason for switching from electricity to kerosene among 83.3% of those who switched to kerosene from electricity with cost of electricity the reason among 16.7%.

Among primary LPG users (*n* = 63), 82.6% and 6.4% previously used kerosene and charcoal respectively as primary cooking fuel within the preceding 5 years. Among participants who switched from kerosene to LPG, lower cost of LPG, health benefits associated with LPG, faster cooking with LPG, convenience in obtaining and using LPG, increase in income and kitchen cleanliness were the reason for switching among 26.9%, 23.1%, 17.3%, 17.3%, 7.7%, 7.7% respectively. Among those who switched from charcoal to LPG, 50% switched due the health benefits of LPG and the rest due convenience in using LPG.

Among primary charcoal users (*n* = 88), 61.3%, 5.7% and 1.1% previously used kerosene, wood, and LPG respectively in the preceding 5 years. Among primary wood users, 50% previously used kerosene and the rest used charcoal. Reasons for switching from kerosene to charcoal or wood were high cost of kerosene, faster cooking with charcoal or wood, health benefits and lack of kitchen space among 78.2%, 16.4%, 5.5% and 1.8% respectively. High cost of LPG was the reason for switching to charcoal among the only user who previously used LPG.

### 3.5. Attitude towards the Use of LPG among Participants

Most participants (77.3%) rated highly the health benefits associated with LPG. Similarly, most participants (65.5%) also reported that they expected a high level of community willingness to use LPG as primary fuel. However, only 26.2% considered that LPG was readily available.

Four hundred and nineteen (91.9%) of non-LPG users expressed willingness to pay for LPG due to the associated health benefits that had become obvious to them by the end of the interview. This comprised of 92%, 100%, 91.8% and 100% of primary charcoal, electricity, kerosene, and wood users respectively. The median amount that they were willing to pay for LPG start up equipment (1 burners and 5-kg refill cylinder) was N4000 ($11) (IQR 3000–5000) which is about half of the current retail price and the median amount they were willing to pay for refill of the 5-kg cylinder was N1000 ($3) (IQR 500–1500) which is also about half of the current retail price.

Among the non-LPG users who were unwilling to pay for LPG (*n* = 37), 29.7%, 13.5%, 13.5%, 5.4% and 2.7% reported risk of fire associated with LPG, high cost of equipment and gas refill, unfamiliarity with use of LPG, spouses’ refusal to use LPG and restrictions by landlords respectively as reasons for unwillingness to pay for LPG. No reason was provided by 37.8% of non-LPG users for their unwillingness to pay for LPG.

## 4. Discussion

Lagos is the largest urban area in Nigeria and over 60% of its populace live in densely populated areas [22]. This study was conducted in one the most densely populated areas in the city. We found that kerosene was used as cooking fuel in nearly all households surveyed either as primary or secondary cooking fuel. In contrast, LPG was used in less than a fifth of the households and charcoal was used in about a third of the households. Stacking of fuels (using more than one cooking fuel) occurred in nearly half of the households. Higher level of education and higher income was significantly associated with LPG use compared to kerosene use while older age (≥60 years) was significantly associated with kerosene vs. LPG use. Health benefits thought to be related to kerosene use was the main reason for switching to kerosene by biomass users while cost of LPG, fear of fire with LPG use, restrictions by landlords and unavailability of LPG were the main reasons for switching from LPG to kerosene. Most LPG users were former kerosene users and cited lower cost of LPG and health benefits associated with LPG use compared to kerosene use as the main reasons for switching from kerosene to LPG. Most non-LPG users were willing to pay for and use LPG. Unwillingness to pay for LPG was mainly due to fear of fire and harm, presumed high cost and unfamiliarity with LPG.

Our findings are consistent with previous reports of the high rate of kerosene use for cooking in Nigeria [10,11,12]. The penchant for kerosene use in Nigeria may have been driven by several years of government subsidy on kerosene which was then considered as a clean cooking fuel for the low and middle-income population [15]. This erroneous belief was also demonstrated in our study which also found that non-LPG users regarded LPG as an expensive fuel choice. However, the reality is that kerosene is a dirty-burning fuel that emits high levels of PM_2.5_, CO and greenhouse gases and has been associated with adverse health and environmental effects [4,8,16,25]. LPG on the other hand is a cleaner burning fuel with several advantages over kerosene; it has been demonstrated to reduce indoor air pollution and greenhouse emissions compared to kerosene [18,19]. LPG emits negligible amounts of black carbon or other pollutants that contribute to global warming and each canister of LPG substituted for kerosene reduces the carbon dioxide emissions by 2.8 kg [26]. LPG use has also been demonstrated to save cooking and cleaning time which has the potential to increase productivity especially among women [18,19].

The Nigerian government spent about $1 billion annually on kerosene subsidy, but in 2016, the government removed kerosene subsidy. The subsidy removal led to increase in the official pump price of kerosene from Naira (N)50 ($0.14) per liter to N83 ($0.23) per liter and led to perennial scarcity and sale of kerosene at unofficial price [15]. Current data from the National Bureau of Statistics indicates that the average price paid by kerosene users as at January 2018 was N284 ($0.79) per liter of kerosene which is about four times the official pump price and most purchases are from independent petroleum product marketers [27]. On the other hand, although there are no strict regulations on the price of LPG, the cost of LPG has remained relatively stable and with a marginal decrease in price between 2017 and 2018. Official data demonstrates that the average cost of refilling a 5-kg gas cylinder decreased by nearly 15% from N2263 ($6.3) in January 2017 to N2190 ($6.1) in January 2018. Similarly, the cost of refill for a 12.5-kg cylinder decreased by 21% from N4453 ($12.4) in January 2017 to N4328 ($12) in January 2018 [28]. Therefore, a policy to promote the transition from kerosene to LPG and scale up the deployment and adoption of LPG should be an obligation for the Nigerian government. The policy should ensure stabilization in the supply of LPG as well as the management and maintenance of LPG cylinders to make it readily and safely available to consumers. This is necessary so as to align with the WHO recommendations and global trends in meeting the SDG 7 that aims to provide affordable, reliable, sustainable, and modern energy for all by 2030 [17,29]. In line with this, a new Nigerian gas policy was approved in 2017 [30]. This policy prioritizes meeting domestic gas obligations accompanied by price monitoring and infrastructural development to increase the availability of LPG and promote its domestic use [30]. The success of this policy however, requires well designed implementation programs to promote the adoption of LPG. This program should include the development of a safe and sustainable model for the domestic LPG market based on cylinder recirculation in which empty cylinders are always exchanged for full ones to ensures safe maintenance of the cylinders by the marketers. The cylinder recirculation program is also a useful approach that may improve LPG adoption by reducing initial cost of outright purchase of cylinders if the households only pay for the gas refills. Indonesia, Brazil, and India for example have demonstrated that a well-crafted intervention program to improve safe and sustainable LPG uptake is feasible and achievable when it is backed by strong government policy and commitment [20,31,32]. The program in Indonesia successfully transitioned about 50 million kerosene users to LPG within four years. This was achieved through a multiphase program that involved first the establishment of infrastructure to ensure adequate and safe supply of LPG, followed by distribution of a filled 3-kg cylinder and stove to entitled households [20]. This was accompanied by education on the safe use and benefits of LPG and identification of local refill stations. Finally, kerosene supply was withdrawn from that locality to ensure the exclusive use of LPG. Evaluation of the effect of this transition program in Indonesia has demonstrated social and economic advantages with potential health benefits for the individuals and the community as well as substantial savings on subsidy by the government. Nearly all participants who adopted LPG in this program reported that LPG was more efficient to cook with and cheaper to use than kerosene [20].

Some of our findings provide guidance towards the design of a successful implementation program aimed at switching Nigerians away from kerosene to LPG. For example, we found that LPG users were significantly more educated than kerosene users and that kerosene users spent significantly more money on fuel than LPG users indicating that the cost of LPG may not be the main driver for non-adoption. Rather it suggests that lack of awareness about the affordability and health benefits associated with LPG and misconceptions regarding safety of LPG may be major drivers for continued kerosene use despite the current high price. This is similar to previous reports from Nigeria and Ghana in which higher socio-economic status which is not only determined by income but encompasses level of education, subjective perception of social status and social class were associated with the adoption of cleaner fuel [33,34,35]. Furthermore, the reasons for switching from LPG to kerosene and for the unwillingness to use LPG in our study also suggest an important role of inadequate information and societal myths in the continued use of kerosene. To further illustrate this, travel time to obtain kerosene and LPG was similar, but about 75% of participants opined that LPG was not readily available. Reassuringly however, the benefits of LPG compared to kerosene use was also demonstrated in our study by current LPG users who switched to LPG from kerosene, charcoal, or wood. LPG users described it as a cleaner and healthier cooking fuel that cooks faster, more cost effective and more convenient to use compared to kerosene. This is similar to reports in the studies in Indonesia and India in which switching to LPG saved cooking time that allowed the women to engage in more productive activities that improved life [20,21]. These findings bring to the fore and support the need for a community wide educational intervention program that highlights the adverse effects of kerosene and the potential benefits of LPG use. This program may independently increase LPG adoption by current kerosene users.

We found that switching to LPG was acceptable to most non-LPG user participants in this study mainly because of the potential health benefits. However, initial cost of equipment and lack of familiarity with the use of LPG were barriers towards the adoption of LPG. The maximum sums that participants in this study were willing to pay for startup equipment and cylinder refills respectively were about half of the current retail prices. The initial cost of equipment (gas burner, tubing, and cylinder) has been recognized as a major barrier to the adoption of LPG in developing countries and steps to mitigate this is fundamental to the success of an implementation program to increase LPG uptake. In Indonesia, the government provided startup equipment for households and over 80% reported that refill of the cylinders was cheap and affordable which resulted in sustainability of the program [20]. During the early years of the LPG transition program in Brazil, the oil companies provided start up equipment and the government provided subsidy on the price of LPG [35]. Today 95% of Brazilian homes use LPG and adequate supply and distribution of LPG is actively regulated by the government [31]. Provision of LPG subsidy in many parts of Latin America has helped transit a majority of the urban poor from use of solid fuels to LPG [36]. In India, the government and major oil companies initiated subsidy programs since 2015 to improve LPG uptake [32,37,38]. The first intervention was the payment of the subsidy directly into the bank accounts of beneficiaries to allow sale of LPG at current market price [32]. This was followed by the “give it up” campaign that encouraged middle class families to give up the subsidy for poorer households [37]. However, in 2017, it was recognized that despite the subsidy, the cost of startup connection was still prohibitive for poorer families to take up LPG. This led to a current program in India called the Pradhan Mantri Ujjwala Yojana (PMUY) which aims to provide free connections to 50 million households by 2019 allowing them to use their subsidies to pay for monthly LPG supply [38].

Ambient air pollution is also an important health hazard, which is influenced by HAP and vice versa [39]. A recent study in India demonstrated that HAP contributes about 24% of the pollutant that lead to ambient air pollution and that this is the largest contributor from any single sector [40]. Outdoor cooking with dirty-burning fuels was common in many households in our study and may substantially contribute to the high levels of ambient PM_2.5_ as reported in the recent study in Lagos [41]. This implies that switching a majority of the populace to cleaner and more efficient cooking fuel may also be an important measure in improving ambient air pollution.

## 5. Conclusions

In conclusion, we found that kerosene is used as cooking fuel in nearly all household in this densely populated area of Lagos with less than a fifth of the households using LPG. LPG use was associated with higher level of education, higher income, and lower age respectively. Monthly expenditure on LPG was significantly lower than for kerosene but kerosene was erroneously considered a cost-effective fuel choice. There was a high willingness by non-LPG users to switch to LPG but perceived safety issues and misconceptions regarding cost as potential barriers to LPG adoption. Our findings highlight the need for the development and implementation of interventions to overcome these barriers so as to scale up LPG adoption. This LPG scale-up program requires strong government policy and commitment from LPG manufacturers and marketers for the expansion of existing LPG distribution infrastructure to increase accessibility, availability, safe deployment, and affordability of LPG to consumers. A community wide educational intervention program that addresses the misconceptions regarding kerosene and LPG use could be an initial effectual and cost-effective approach to improve LPG adoption. It is also likely to be fundamental to the success of other additional LPG scale-up interventions. We recommend that such an approach should be formally evaluated through a well-designed community-based intervention study.

## Figures and Tables

**Table 1:** Socio-demographic characteristics of all participants.

Characteristic	Description *n* = 519
Gender	
Female (%)	420 (80.9)
Status in household	
Wife (%)	269 (51.8)
Female head of household (%)	139 (26.8)
Male head of household (%)	92 (17.9)
Representative of household (%)	19 (3.7)
Main income earner	
Yes (%)	264 (50.9)
Main household spender	
Yes (%)	348 (67.1)
Decides choice of cooking fuel	
Yes (%)	444 (85.5)
Level of education	
None (%)	55 (10.6)
Primary (%)	100 (19.3)
Junior secondary (%)	48 (9.3)
Senior secondary (%)	220 (42.4)
University/polytechnic (%)	96 (18.5)
Marital status	
Married/co habiting (%)	402 (77.5)
Single (%)	48 (9.2)
Widowed (%)	45 (8.7)
Separated/divorced (%)	24 (4.7)
Age	
Mean (SD)	39.2 (12.2)
Number in household	
Total number, Median (IQR)	4 (3–6)
Number of adults in household, Median (IQR)	2 (2–3)
Type of housing	
Single room with shared amenities (%)	456 (87.9)
Three rooms with shared amenities (%)	1 (0.2)
Single room with personal amenities (%)	29 (5.6)
Two bedrooms flat (%)	24 (4.6)
Three bedrooms flat (%)	7 (1.3)
Five bedrooms apartment (%)	2 (0.4)
Home ownership	
No (%)	466 (89.8)

SD = Standard deviation, IQR = interquartile range, shared or personal amenities include kitchens, bathrooms, and toilets.

**Table 2:** Choice of primary and secondary household cooking fuels.

Fuel Type Use in Households	Primary Fuel *n* (%)	Secondary Fuel *n* (%)	Total *n* (%)
Kerosene	364 (70.1)	111 (21.4)	475 (90.5)
Charcoal	88 (17)	71 (13.7)	159 (30.6)
LPG	63 (12.1)	23 (4.4)	86 (16.6)
Electricity	2 (0.4)	20 (3.9)	22 (4.2)
Wood	2 (0.4)	5 (10)	7 (1.3)

LPG = Liquefied Petroleum Gas. No participant used coal, dung, agricultural waste or saw dust for household cooking.

**Table 3:** Location of kitchen among primary fuel users.

Type of Primary Fuel	Kitchen Location		
	Indoor Separate from Living and Sleeping Area (%)	Indoor Within Living and Sleeping Area (%)	Outdoor (%)
Kerosene	140 (38.4)	64 (17.6)	160 (44)
Charcoal	21 (23.8)	16 (18.2)	51 (58)
LPG	38 (60.3)	8 (12.7)	17 (27)
Electricity	2 (100)	0	0
Wood	0	0	2 (100)

**Table 4:** Comparison of the knowledge of harmful health effects associated with cooking with biomass and kerosene.

Harmful Health Effect	Participants with Knowledge of Association with Biomass Use (%)	Participants with Knowledge of Association with Kerosene Use (%)	Statistics
Pneumonia	245 (47.2)	76 (14.6)	X^2^ = 100.9, *p* < 0.001
Tuberculosis	309 (59.5)	80 (15.4)	X^2^ = 82.7, *p* < 0.001
Breathing problems	429 (82.7)	77 (14.8)	X^2^ = 59.7, *p* < 0.001
Lung cancer	269 (51.8)	111 (21.4)	X^2^ = 102, *p* < 0.001
Asthma	376 (72.4)	93 (17.9)	X^2^ = 114.9, *p* < 0.001
Bronchitis	333 (64.2)	80 (15.4)	X^2^ = 82.6, *p* < 0.001
Running nose	403 (77.6)	104 (20)	X^2^ = 62.5, *p* < 0.001
Watery eyes	443 (85.4)	133 (25.7)	X^2^ = 64.0, *p* < 0.001
Any health effect	465 (89.6)	115 (29.9)	X^2^ = 14.0, *p* = 0.001

**Table 5:** Association between fuel types and selected variables.

Variable	Primary Fuel					
	Kerosene, *n* = 364	Charcoal, *n* = 88	liquefied Petroleum Gas (LPG), *n* = 63	Electricity, *n* = 2	Wood, *n* = 2	Statistics
Level of education						
None or Primary (%)	104 (28.6)	43 (48.9)	7 (11.1)	0	1 (50)	X^2^ = 52.2, *p* = 0.004
Secondary (%)	192 (52.7)	37 (42)	36 (57.1)	1 (50)	1 (50)	
Post-secondary (%)	68 (18.7)	8 (9.1)	20 (31.8)	1 (50)	0	
Age group (years)						
<20 (%)	3 (0.8)	2 (2.3)	0	0	0	X^2^ = 24.7, *p* = 0.42
20–29 (%)	78 (21.4)	18 (20.5)	21 (33.3)	0	1 (50)	
30–39 (%)	122 (33.5)	28 (31.8)	15 (23.8)	1 (50)	1 (50)	
40–49 (%)	81 (22.3)	24 (27.3)	19 (30.2)	0	0	
50–59 (%)	41 (11.3)	6 (6.8)	8 (12.7)	1 (50)	0	
60–69 (%)	30 (8.2)	9 (10.2)	0	0	0	
≥70 (%)	9 (2.5)	1 (1.1)	0	0	0	
Income (Naira)						
<20,000	81 (22.3)	20 (22.7)	10 (15.9)	1 (50)	1 (50)	X^2^ = 40.8, *p* = 0.06
20,000–40,000	111 (30.5)	42 (47.7)	20 (31.7)	0	1 (50)	
41,000–60,000	101 (27.7)	16 (18.2)	16 (25.4)	0	0	
61,000–80,000	31 (8.5)	5 (5.7)	6 (9.5)	1 (50)	0	
81,000–100,000	11 (3.0)	1 (1.1)	1 (1.6)	0	0	
>100,000	29 (8.0)	4 (4.5)	10 (15.9)	0	0	
Method of preparation of staple food influences fuel choice (%)	28 (7.7)	15 (17)	10(15.9)	0	0	X^2^ = 16.07, *p* = 0.04
Cooking time for preparation of staple food influences fuel choice (%)	30 (8.2)	13 (14.8)	10 (15.9)	0	0	X^2^ = 6.19, *p* = 0.19
Time travelled to obtain cooking fuel in minutes						
<15	271 (74.5)	67 (76.1)	39 (61.9)	Not applicable	2 (100)	X^2^ = 12.73, *p* = 0.12
15–30	76 (20.9)	17 (19.3)	19 (30.2)			
>30	17 (4.7)	4 (4.5)	5 (7.9)			
Monthly expenditure on cooking fuel in Nigerian Naira (Mean ± SD)	2581.6 ± 1407.5	2780.1 ± 2248.6	2169.0 ± 1507.0	1000.0 ± 707.1	2450.0 ± 70.7	*F* = 1.58, *p* = 0.004
Knowledge of any harmful health effect associated with biomass use (%)	328 (90.1)	78 (88.6)	56 (88.9)	1 (50)	2 (100)	X^2^ = 11.12, *p* = 0.20
Knowledge of any harmful health effect associated with kerosene use	112 (30.8)	18 (20.5)	22 (34.9)	2 (100)	1 (50)	X^2^ = 9.72, *p* = 0.05

SD = Standard deviation.

## Supplementary Materials

Supplementary File 1

## References

[1] World Health Organization (2014). Burden of Disease from Household Air Pollution, 2012.

[2] Burnett R.T., Pope A.C., Ezzati M., Olives C., Lim S.S., Mehta S., Shin H.H., Singh G., Hubbell B., Brauer M. (2014). An integrated risk function for estimating the global burden of disease attributable to ambient fine particulate matter exposure. Environ. Health Perspect..

[3] World Health Organization (2014). Indoor Air Quality Guidelines: Household Fuel Combustion.

[4] Lam N.L., Smith K.R., Gauthier A., Bates M.N. (2012). Kerosene: A review of household uses and their hazards in low-and middle-income countries. J. Toxicol. Environ. Health.

[5] Pokhrel A.K., Bates M.N., Verma S.C., Joshi S.H., Sreramareddy C.T., Smith K.R. (2010). Tuberculosis and indoor biomass and kerosene use in Nepal: A case-control study. Environ. Health Perspect..

[6] Choi J.Y., Baumgartner J., Harnden S., Alexander B.H., Town R.J., D’Souza J., Ramachandran G. (2014). Increased risk of respiratory illness associated with kerosene fuel use among women and children in urban Bangalore. Indian J. Occup. Environ. Med..

[7] Venn A.J., Yemaneberhan H., Bekele Z., Lewis S.A., Parry E., Britton A. (2001). Increased risk of allergy associated with the use of kerosene fuel in the home. Am. J. Respir. Crit. Care Med..

[8] Bates M.N., Chandyo R.K., Valentiner-Branth P., Pokhrel A.K., Mathisen M., Basnet S., Shrestha P.S., Strand T.A., Smith K.R. (2013). Acute lower respiratory infection in childhood and household fuel use in Bhaktapur, Nepal. Environ. Health Perspect..

[9] Adeniji B.A., Ana G.R.E.E., Adedokun B.O., Ige O.I. (2015). Exposure to emissions from kerosene cooking stoves and the pulmonary health status of women in Olorunda community, Ibadan, Nigeria. J. Environ. Prot..

[10] Ohimain I.E. (2012). The benefits and potential impacts of household cooking fuel substitution with bio-ethanol produced from cassava feedstock in Nigeria. Energy Sustain. Dev..

[11] Desalu O.O., Ojo O.O., Ariyibi E.K., Kolawole T.F., Ogunleye A.I. (2012). A community survey of the pattern and determinants of household sources of energy for cooking in rural and urban south western, Nigeria. Pan Afr. Med. J..

[12] Adegbemi B.O., Adegbemi O.O., Olalekan A.J., Babatunde O.O. (2013). Energy consumption and Nigerian economic growth: An empirical analysis. Eur. Sci. J..

[13] National Population Commission—NPC/Nigeria and ICF International (2014). Nigeria Demographic and Health Survey 2013.

[14] Oyekale A.S., Dare A.M., Olugbire O.O. (2012). Assessment of rural households cooking energy choice during kerosene subsidy in Nigeria: A case study of Oluyole Local Government Area of Oyo State. Afr. J. Agric. Res..

[15] Ohaeri V., Adeyinka T. (2016). Policy Brief: Kerosene subsidy reform and the burden of supply. Gend. Energy Policy.

[16] Bates M.N., Pokhrel A.K., Chandyo R.K., Valentiner-Branth P., Mathisen M., Basnet S., Strand T.A., Burnett R.T., Smith K.R. (2018). Kitchen PM_2.5_ concentrations and child acute lower respiratory infection in Bhaktapur, Nepal: The importance of fuel type. Environ. Res..

[17] United Nations Development Program Sustainable Development Goals. Goal 7: Affordable and Clean Energy. http://www.undp.org/content/undp/en/home/sustainable-development-goals/goal-7-affordable-and-clean-energy.html.

[18] Bruce N.G., Aunan K., Rehfuess E.A. (2017). Liquefied Petroleum Gas as a Clean Cooking Fuel for Developing Countries: Implications for Climate, Forests, and Affordability.

[19] Van Leeuwen R., Evans A., Hyseni B. (2017). Increasing the Use of Liquefied Petroleum Gas in Cooking in Developing Countries.

[20] Budya H., Arofat H.Y. (2011). Providing cleaner energy access in Indonesia through the megaproject of kerosene conversion to LPG. Energy Policy.

[21] Nautiyal S. (2013). Transition from Wood Fuel to LPG and Its Impact on Energy Conservation and Health in the Central Himalayas. J. Mt. Sci..

[22] The Largest Cities in the World by Land Area, Population and Density. http://www.citymayors.com/statistics/largest-cities-density-125.html.

[23] World Population Review Lagos Population 2017. http://worldpopulationreview.com/world-cities/lagos-population/.

[24] Federal Republic of Nigeria Official Gazette (2009). Legal Notice on Publication of the Details of the Breakdown of the National and State Official Totals 2006 Census.

[25] Khalequzzaman M., Kamijima M., Sakai K., Chowdhury N.A., Hamajima N., Nakajima T. (2007). Indoor air pollution and its impact on children under five years old in Bangladesh. Indoor Air.

[26] Greenworks Asia (2008). Calculation on Emission Reduction from LPG Conversion Program, Jakarta. http://www.greenworksasia.com/experiences.html.

[27] National Bureau of Statistics National Household Kerosene Price Watch-January 2018. http://nigeria.opendataforafrica.org/attyre/national-household-kerosene-price-watch-january-2018.

[28] National Bureau of Statistics National Cooking Gas Price Watch-January 2018. http://nigeria.opendataforafrica.org/oieqsne/cooking-gas-price-watch-january-2018.

[29] World Health Organization (2016). Burning Opportunity: Clean Household Energy for Health, Sustainable Development, and Wellbeing of Women and Children.

[30] Federal Republic of Nigeria, Ministry of Petroleum Resources (2017). National Gas Policy, Nigerian Government Policy and Action. http://www.petroleumindustrybill.com/wp-content/uploads/2017/06/National-Gas-Policy-Approved-By-FEC-in-June-2017.pdf.

[31] Da Silva J.L.M. (2007). Transformações no espaço doméstico: O fogão a gás e a cozinha paulistana, 1870–1930. An. Mus. Paul. História E Cult. Mater..

[32] Sharma S. (2014). Subsidies to Liquefied Petroleum Gas in India: An Assessment of the Direct Benefit Transfer in Mysore.

[33] Ifegbesan A.I., Rampedi I.T., Annegarn H.J. (2016). Nigerian households’ cooking energy use, determinants of choice, and some implications for human health and environmental sustainability. Habitat. Int..

[34] Karimu A., Tei Mensah A., Adu G. (2016). Who adopts LPG as the main cooking fuel and why? Empirical evidence on Ghana based on national survey. World Dev..

[35] American Psychological Association Education and Socioeconomic Status Fact Sheet. http://www.apa.org/pi/ses/resources/publications/index.aspx.

[36] Troncoso K., da Silva A.S. (2007). LPG fuel subsidies in Latin America and the use of solid fuels to cook. Energy Policy.

[37] Smith K.R., Sagar A.D. (2016). LPG subsidy: Give it up, if you haven’t. Finance Times of India.

[38] Smith K.R., Debroy B., Malik A. (2017). The Indian LPG Programmes: Globally Pioneering Initiatives. India at 70, Modi at 3: Reform, Perform to Transform India.

[39] Mannucci P.M., Franchini M. (2017). Health effects of ambient Air pollution in developing countries. Int. J. Environ. Res. Public Health.

[40] GBD MAPS Working Group (2018). Burden of Disease Attributable to Major Air Pollution Sources in India.

[41] Njoku K.L., Rumide T.J., Akinola M.O., Adesuyi A.A., Jolaoso A.O. (2016). Ambient air quality monitoring in metropolitan city of Lagos, Nigeria. J. Appl. Sci. Environ. Manag..

